# Development of a cohort multiple randomized clinical trial to test an integrated system of sensors and multimedia monitors technology, for stroke rehabilitation: the ROOMMATE study protocol

**DOI:** 10.3389/fneur.2025.1568728

**Published:** 2025-10-14

**Authors:** Stefano Doronzio, Stephanie Jansen-Kosterink, Margherita Tesi, Chiara Castagnoli, Chiara Pedrini, Tommaso Ciapetti, Mario De Marco, Michele Piazzini, Julieta Giacani, Ileana Ciobanu, Mihai Berteanu, Laura Fiorini, Erika Rovini, Filippo Cavallo, Francesco Agnoloni, Marco Baccini, Francesca Cecchi

**Affiliations:** ^1^Neuromoter Research Unit, IRCCS Fondazione Don Carlo Gnocchi, Firenze, Italy; ^2^Department of Experimental and Clinical Medicine, University of Florence, Firenze, Italy; ^3^Roessingh Research and Development, Enschede, Netherlands; ^4^School of Specialization in Physical and Rehabilitation Medicine, University of Florence, Florence, Italy; ^5^Department of Physical and Rehabilitation Medicine, Carol Davila University of Medicine and Pharmacy, Elias University Emergency Hospital, Bucharest, Romania; ^6^Department Industrial Engineering, University of Florence, Florence, Italy; ^7^Medea S.r.l., Florence, Italy

**Keywords:** stroke, digital ecosystem, enriched environment, cohort multiple randomized controlled trial, digital health adoption

## Abstract

**Background:**

Stroke remains a leading cause of disability globally, creating significant challenges for healthcare systems. Early intensive rehabilitation is recommended (NICE2023) for functional recovery; but standard therapy may not cover all patients’ needs. Stroke survivors should continue active task practice outside of scheduled therapy sessions (self-directed, or semi-supervised by family and caregivers), possibly by sustainable technological solutions. However, most technology-based ecosystems are not designed for healthcare. To test a digital stroke rehab ecosystem Randomized Clinical Trials (RCTs) are the gold standard, but present ethical and logistical challenges, particularly in blinding and participant adherence. This study protocol (ClinicalTrials.gov; NCT06728020; March 21 st, 2025) employs a cohort multiple RCT (cmRCT) innovative design, suitable for comparing usual care to interventions in studies with multiple interventions: cmRCT enrols a large observational cohort, allowing random selection of participants for individual trials rather than random allocation for all subjects, alongside a patient-centered approach to information and consent.

**Methods:**

A certified device including a multimedia monitor with virtual reality cognitive and motor rehabilitation exercises (VRRS) will be enriched with educational videos and rehabilitation contents, developed by co-creation involving stroke patients, caregivers and rehabilitation professionals (VRRS1). A dynamic cohort of post-acute stroke inpatients will then be prospectively enrolled in a cmRCT: first, the VRRS1 will be randomly proposed to and tested with 70 subjects, while 70 other eligible patients will be randomized to Usual Care (UC) - controls (ROOMMATE 1st); then, the VRRS1, integrated with a set of inertial sensors (BMR4ROOMMATE) (VRRS2), will be tested in a pilot RCT on 30 patients (while 30 other eligible patients will be randomized as controls - ROOMMATE 2nd). The primary outcome will be the Modified Barthel Index, while secondary outcomes will include measures of motor and cognitive functions, as well as feasibility, usability, and device wearability.

**Conclusion:**

By combining sensor-based assessment, expert coaching on digital literacy, and the active involvement of patients, caregivers, and healthcare professionals ROOMMATE aims to co-create an innovative digital ecosystem for Stroke rehabilitation. By rigorously verifying its impact by a cmRCT, it seeks evidence to enhance stroke recovery beyond usual care.

## 1 Introduction

Neurological disorders are a leading cause of disability worldwide, stroke being the most prevalent. In 2017, there were 1.12 million incident strokes in the European Union and 9.53 million stroke survivors. Also, stroke prevalence is estimated to increase by 27% between 2017 and 2047, due to population aging and to improved survival rates (1), and stroke-related disability is estimated to have a parallel increase (2).

Early intensive rehabilitation and an enriched environment are important promoters of neuroplasticity and functional recovery after stroke. However, even after intensive rehabilitation, stroke survivors often achieve limited recovery in cognitive and motor functions, activities and participation (2). Stroke patients’ associations report (3) that patients feel abandoned, with unmet needs including disease-related information, physical recovery and activity/participation, social environmental resources, and psycho-emotional support. Indeed, in typical hospital settings patients lack structured planned activities for long hours outside of scheduled rehabilitation activities, and frequently due to economic and organizational constraints (3) they also tend to be disengaged and isolated from their families for most of the day.

Recent updates from the 2023 NICE stroke guidelines further emphasize that people after stroke should be offered needs-based rehabilitation for at least 3 h per day, on at least 5 days per week, delivered through a multidisciplinary approach including physiotherapy, occupational therapy, and speech and language therapy (4, 5), but also highlights the importance of encouraging stroke survivors to continue active task practice outside of scheduled therapy sessions, through self-directed or semi-supervised practice, and with the involvement of family and caregivers whenever appropriate (5).

Technology provides opportunities to optimize rehabilitation services by providing sustainable, intensive interventions addressing more functioning domains at once, but its impact on rehabilitation outcome and the cost-effectiveness of technology-based rehabilitation interventions on stroke patients require further evidence (4, 6). Further, the acceptance of rehabilitation technologies is mainly hindered by the limited health and digital literacy of potential users. Almost 50% of European citizens have low levels of health literacy, and even lower levels of digital literacy, and structured coaching programs to support different end users towards adoption of technological components are lacking (7). Finally, most of the digital health ecosystems have been developed over existing platforms, created and implemented for purposes not specifically directed to care and services provision; these ecosystems generally do not take into account standards required for medical technologies (8) nor the lack of skills of real users (9).

Previous studies highlight that co-creation in stroke rehabilitation is still in a rudimentary phase, with inconsistent terminology and diverse methodologies (10, 11). Also, digital therapies have shown promise in improving clinical outcomes, patient empowerment and quality of life; however, challenges remain in extending benefits to those with severe neurological impairments and in addressing digital health equity (12). While numerous subsystems, such as telehealth, exergaming, virtual reality, robotics, mobile health apps and digital support platforms have been developed, very few examples of health technology ecosystems exist (13), where these components integrate to support multiple clinical processes, shifting toward, patient-centered care, with key implications for rehabilitation quality and resource allocation in digital health. Indeed, the assessment of these systems should consider several domains, i.e., feasibility, usability, acceptability, user’s experience, effectiveness and cost–benefit ratio. Regarding the last two, a relevant issue is to identify a study design that allows the pragmatic investigation of the different components of the ecosystem. The NICE 2023 guidelines further acknowledge that telehealth services may be used either as an adjunct or as an alternative to in-person therapy, particularly for patients unable to access community rehabilitation. Importantly, delivering interventions via telehealth should be restricted to approaches with proven benefits (5, 14). This provides strong support for testing bedside telerehabilitation within neurorehabilitation units as part of structured, needs-based care.

In clinical research, the Randomized Controlled Trial (RCT) study design is considered the gold standard approach to verify the effectiveness of an intervention (15). On the other hand, the feasibility of RCTs in physical and rehabilitation medicine raises ethical as well as practical issues. Since RCTs require full disclosure of the protocol, problems with this study design also concern patient preferences, which can be a major issue for adherence, particularly when participants are not blind to group allocation. Patients may be less motivated to participate if they suspect or know for sure that they will not receive the experimental treatment. This may lead to refusal to participate, withdrawal or less compliance with the protocol. The cohort multiple RCT (cmRCT) is an innovative design that attempts to overcome these issues related to traditional RCT (16). The key features of this design are: (I) recruitment of a large observational cohort of patients with the condition of interest, providing consent to the observational study and to be eventually enrolled in multiple RCTs; (II) regular measurement of outcomes for the whole cohort (III); capacity for multiple randomized controlled trials over time. For each randomized controlled trial, (IV) identification of all eligible patients in the whole cohort (NA); (V) random selection of some patients (nA) from all eligible patients in the cohort, who are then offered the trial intervention; (VI) comparison of the outcomes in randomly selected patients (B) with the outcomes in eligible patients not randomly selected; that is, those receiving usual care (UC) (NA − B); (VII) “Patient centred” informed consent to the experimental intervention; that is, the process of obtaining patient information and consent aims to replicate that in real world routine health care, when you actually will provide the intervention you propose if the participant accepts. The cohort multiple RCT allows the simultaneous evaluation of the effectiveness of several interventions (16). In cmRCTs, ethical requirements are not violated due to the fact that all cohort subjects are informed about the study design at the time of enrolment in the study cohort, when they are asked for informed consent to participate and also agree to be either contacted for receiving experimental interventions or to serve as controls without additional notification (16).

In this context, the ROOMMATE project, co-funded by the European Union within the “Transforming Health Care Systems” call (GA N° 101,095,654 of the Horizon Europe Research and Innovation Program), seeks to address these challenges, by involving all stakeholders (patients, caregivers, health professionals) in the co-creation of a technology supported ecosystem to optimize stroke rehabilitation, and testing it against usual care (UC).

Our hypothesis is that the implementation of such ecosystem will be feasible, usable and appreciated by users. We also hypothesize that it will improve functional recovery in stroke survivors undergoing post-acute intensive rehabilitation, compared to usual care (UC).

## 2 Materials and methods

### 2.1 Study design

The registration on ClinicalTrials.gov has been completed and the study has been reviewed obtaining the identification number “NCT06728020.” This study was prospectively registered as an international multicenter no-profit cmRCT and developed according to the Standard Protocol Items: Recommendations for Interventional Trials (SPIRIT) checklist (17), as detailed is in Supplementary material.

First, we will develop educational videos and rehabilitation contents, co-created with patients, caregivers and rehabilitation professionals, and introduce them into a certified device a multimedia system that already includes virtual reality cognitive and motor rehabilitation exercises (VRRS1). VRRS1 will be tested in a first RCT (ROOMMATE 1st), providing expert coaching on its use throughout the intervention. Subsequently, VRRS1 will be integrated with a set of inertial sensors (BMR4ROOMMATE) and this integrated system (VRRS2) will be tested in a second pilot RCT (ROOMMATE 2nd).

### 2.2 Study setting

The study will be conducted at the Don Carlo Gnocchi Foundation (FDG) in Florence and the Elias University Emergency Hospital (EUEH) in Bucharest. FDG is the coordinator clinical center, with dedicated facilities and multidisciplinary expertise in post-stroke rehabilitation and innovative digital rehabilitation approaches. EUEH is one of Romania’s major university hospitals, hosting specialized units for acute and post-acute stroke care, and expertise in health technologies co-creation.

### 2.3 Participants

Two hundred persons with stroke outcomes admitted to the neurorehabilitation wards of (100 participants each) will be enrolled in the study cohort. All consecutive patients meeting the eligibility criteria will be included until the prospected sample size is reached. Both clinical sites applied the same eligibility criteria to ensure consistency in patient selection. The enrollment was competitive.

Inclusion criteria:

- age ≥ 18 years;
- outcome of ischaemic or haemorrhagic stroke in subacute phase (< 3 months from the event);
- the ability to use devices included in the digital ecosystem independently or in the presence of a caregiver who can assist the patient in using the system;
- willingness to participate in the project, with informed consent signed by the patient or by his/her immediate relatives/legal guardians, when necessary.
- Exclusion criteria:
- severe uncorrectable visual and/or hearing impairment;
- Skin or other peripheral lesions that prevent the inertial sensors from being worn;
- Clinical signs of medical instability, defined by a score greater than zero on the Clinical Instability Score (CIS) (18).

### 2.4 Devices description

**Figure 1:**
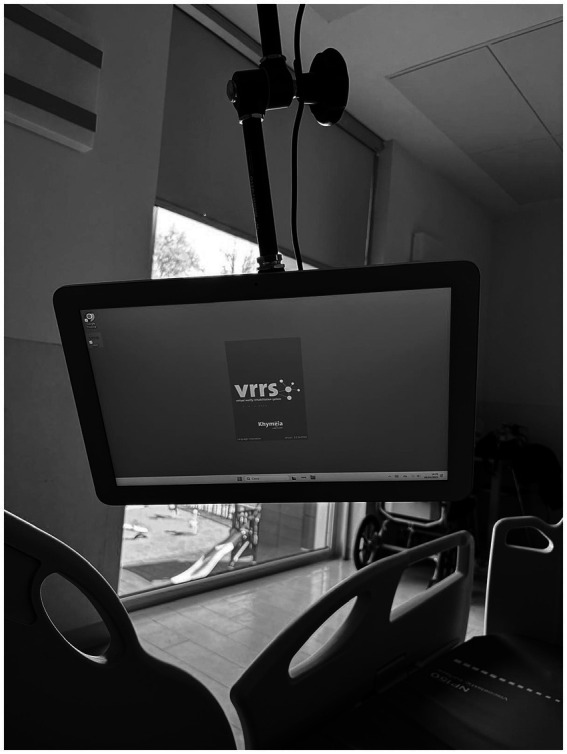
VRRS home kit cognitive.

The digital ecosystem will consist of a multimedia monitor apparatus, named Virtual Reality Rehabilitation System (VRRS). In particular, VRRS Home Kit Cognitive produced by Khymeia Group. VRRS is a CE-certified device for remote cognitive and speech therapy, supporting online therapist-led sessions and offline patient-guided exercises with real-time feedback. The system is tablet-based and provides modules for motor, cognitive, and language rehabilitation. It offers virtual rehabilitation, task-based feedback, automated performance tracking, and session reports, and enables the design of a personalized treatment plan that can be shared with the patient, and it is characterized by a user-friendly interface along with the ability to select different levels of difficulty when performing exercises. Within the ROOMMATE project, the device will be integrated with original educational and rehabilitation contents, co-created within the project’s framework, providing a bedside rehabilitation station (Figure 1) The device is classified as a Class I non-invasive medical device (MDR 2017/745).

**Figure 2:**
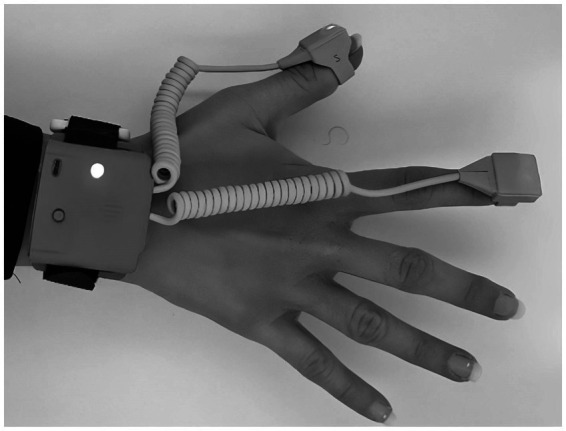
BMR4ROOMMATE wearable device. It is composed of 3 inertial measurement units (IMUs) one placed on the wrist and two placed on the fingers.

The BMR4ROOMMATE system is classified as a Class I non-invasive medical device (MDR 2017/745) specifically designed for quantitative motion analysis in neurological and orthopedic research. Developed by the University of Florence, Department of Industrial Engineering (DIEF) within the Assistive BioRobotics Joint Lab, it combines wearable inertial sensors and a depth camera to provide detailed biomechanical assessments. BMR4ROOMMATE includes two sensorized rings and a wrist bracelet that together measure upper limb motion, capturing acceleration, angular velocity, and magnetic field data. Each unit integrates a 9-axis inertial sensor (accelerometer, gyroscope, and magnetometer) alongside an ARM Cortex-M3 microcontroller. Data from the rings and bracelet are synchronized via CAN-BUS protocol and transmitted to a PC through Bluetooth (Rigado BMD-350). The system acquires data at 100 Hz, ensuring high-resolution analysis. To accommodate different hand sizes, five interchangeable ring sizes are available (XS to XL). For the lower limb, the SensFoot module—housing the same sensor and processor—is anchored to the patient’s shoe, enabling gait and postural assessments without restricting foot movement. An Intel RealSense RGB-D camera complements the wearable sensors by providing three-dimensional depth data on patient movements, enhancing the overall biomechanical evaluation. The camera operates locally with no remote data exchange. The BMR4ROOMMATE device does not interfere with patient performance and is easy to wear, requiring only a standardized fitting procedure. The system is provided free of charge to participating clinical centers (Figure 2).

### 2.5 Procedure

**Figure 3:**
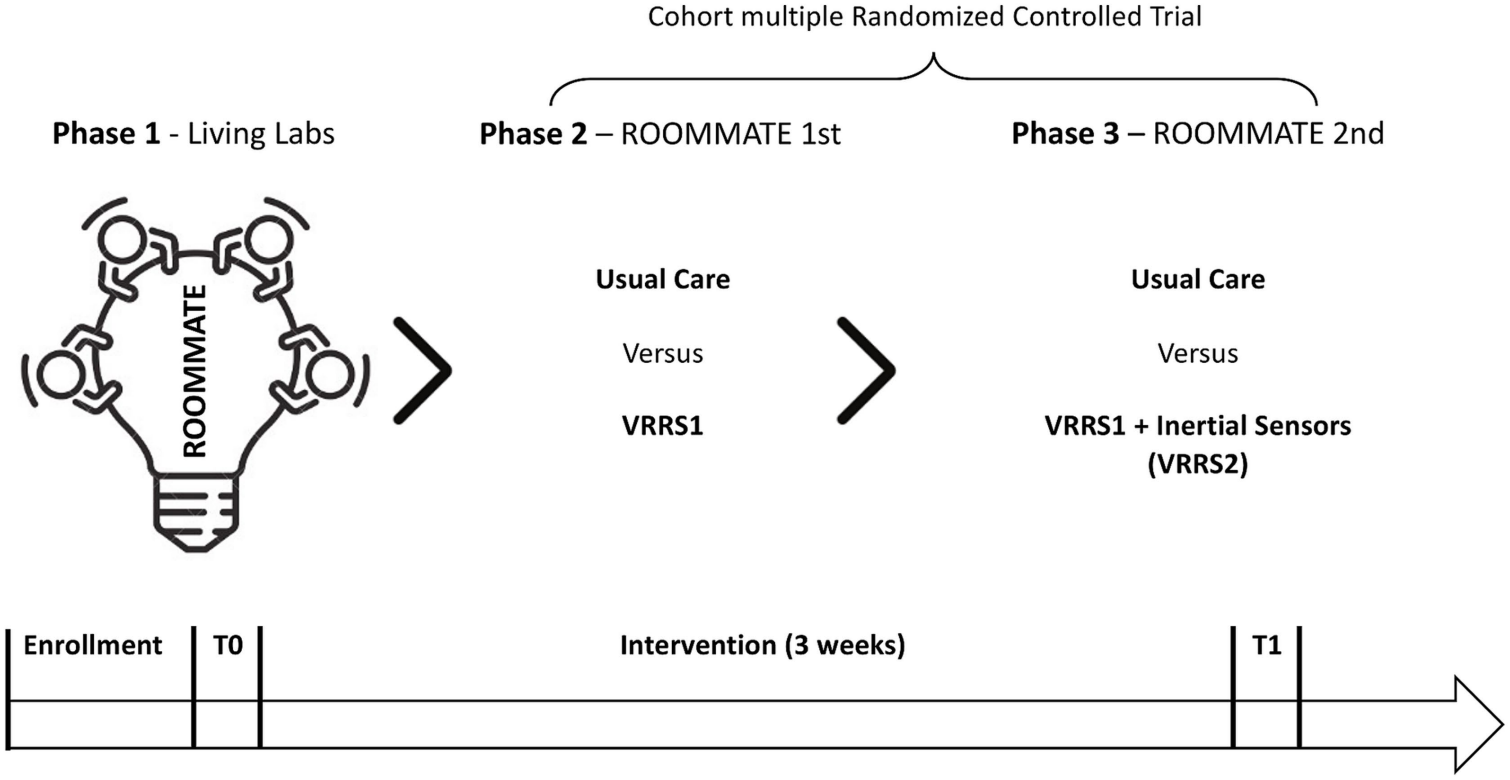
Phases of the clinical protocol.

The study will be carried out in the neurorehabilitation wards of the two clinical centers, involving inpatients undergoing subacute stroke rehabilitation. In detail, in line with the innovative vision of the project, the VRRS Home Kit Cognitive system, including the co-created educational and rehabilitation contents, will be available to patients and caregivers directly in their rooms. In particular, the device will be mounted at the patient’s bedside with an extendable arm, allowing the screen to be positioned in the most comfortable orientation for each patient, as shown in Figure 1. The BMR4ROOMMATE devices, on the other hand, will be used by the project’ research team, to provide a sensorized motor assessment of upper limb at different time points point during the rehabilitation and correlate it with the data on the patient’s motor performance in the VRRS upper limb exergames. The phases of the study are shown in Figure 3.

Phase 1: before starting to enroll participants, a preliminary phase (phase 1 of the ROOMMATE project) is planned to co-create the digital ecosystem and train end-users in its use. First, questionnaires will be administered, and semi-structured interviews, focus groups and workshops will be conducted with all the relevant stakeholders (rehabilitation healthcare professionals, persons with post-stroke disabilities, caregivers), according to a standardized co-creation methodology. Iterative feedback from these participative activities will be used to develop and test the educational and rehabilitation contents and tailor them to opinions, needs, preferences and values in regards to rehabilitation of relevant stakeholders (19, 20). Afterwards, all different categories of stakeholders will test the proposed technologies, in different stages of development of the solution, from wireframes to functional prototypes, and will be asked their feedback on their acceptability and usability in Living Labs (LLs), where they will be trained in the use of the system by e-health coaches (e-HC), chosen among the bedside researchers (physiotherapists, occupational therapists, speech therapists, psychologists, nurses) of the clinical sites, and trained for this task by a two-week course with a final examination on e-health and digital technologies mastership and on coaching skills. The focus groups will follow a standardized co-creation methodology (21), with structured discussions aimed at gathering insights from patients, caregivers, and professionals about unmet needs after a stroke, in order to shape the content of the digital ecosystem. During these meetings, however, VRRS devices will not be experienced by participants. Instead, in the living labs, participants will interact with VRRS1 to assess its usability and functionalities. The VRRS2, which incorporates the BMR4ROOMMATE inertial sensor for evaluation purposes, will not be included in these sessions. Phase 1 ensures that the digital ecosystem is shaped by real user needs before further testing. The qualitative results of the usability testing will be used to refine the user-technology interfaces and to create appropriate workflows to improve user experience and rehabilitation outcomes. The LL activities will be organized in the occupational therapy (OT) labs at both clinical sites, where techniques such as thinking aloud and discussions on specific aspects will be used, while the research team members offer cognitive walkthroughs and participants explore the workflows provided by the technologies on their own. Time and the success rate of participants while exploring and solving tasks using the technologies will be recorded to provide technology developers with valuable feedback in order to adapt the interfaces and the navigation to improve usability for the target populations.

In Phase 2 and Phase 3, the digital system developed in phase 1 will be tested and compared to UC. Two slightly different technologies assemblies, named VRRS1 and VRRS2, both aimed at building a technology supported enriched environment, will be studied in succession. VRRS1 includes all the motor, cognitive and speech therapeutic modalities embedded in the original VRRS plus the informative and rehabilitation contents co-created in the first phase of the project; VRRS 2 includes all the features of VRRS1, that will be integrated to the BMR4ROOMMATE devices, providing a specifically developed sensorized motor assessment of the upper limb.

In phase 2, the VRRS1 will be tested in a randomized controlled trial enrolling a dynamic cohort of 140 participants, by a competitive recruitment in the two Clinical Centres (FDG in Florence, EUEH in Bucharest) through the systematic assessment for eligibility of all patients addressed to either centre for post-stroke inpatient rehabilitation. Eligible patients who will sign the informed consent will be consecutively recruited in either centre. Half of them will be randomly assigned to be proposed to receive the intervention VRRS1 in addition to usual care, and to be enrolled after signing another informed consent, while the other 70 will serve as controls, receiving only usual care. This first RCT is called ROOMMATE 1rst. In parallel to phase 2, the technical partners will carry out the integration of the VRRS with the MB4ROOMMATE inertial sensor.

At the end of phase 2, in both Clinical Centres the cmRCT recruitment will continue as before to enroll a dynamic cohort of at least 60 subjects in total; half of them will be randomly extracted to be proposed to participate in a pilot RCT (ROOMMATE 2nd) to study the effects of the integrated system VRRS2 (phase 3), and enrolled if providing informed consent, while the other 30 will serve as controls receiving usual care.

The VRRS system includes the proprietary content developed by Khymeia, consisting in a structured set of motor and cognitive exercises, each targeting specific rehabilitation objectives:

- Motor Deficit exercises: these exercises address impairments in movement and coordination and are designed to improve strength, range of motion and coordination; examples include arm elevation, reaching and grasping activities, trunk flexion-extension, ankle dorsal and plantar flexion;
- Cognitive Deficit exercises: these focus on visuospatial abilities, attention, and executive functions, using interactive tasks that require target tracking, hand-eye coordination, and memory recall.

Additionally, alongside this content, the VRRS1 system will incorporate rehabilitation and educational content co-created during Phase 1 of the project with key stakeholders, including patients, caregivers, and healthcare professionals. The exercises provided by the VRRS1 and later by the VRRS2 system will be chosen and performed according to the personalized prescription of the therapists taking care of the patient during his/her stay.

VRRS1 (and later VRRS2) will be tested as a bedside rehabilitation station and made available to patients, caregivers and rehabilitation professionals (physiotherapists, speech and language therapists, occupational therapists). Strategies to improve adherence to intervention protocols, and procedures for monitoring adherence, included the availability of an e-HealthCoach (e-HC) to support patients, caregivers, and therapists, fostering innovation adoption and promptly addressing any system malfunctions. Criteria for discontinuing or modifying allocated interventions included withdrawal of informed consent, clinical worsening, or any adverse effects attributable to the intervention. In the trial, no treatments deviating from usual care (UC), as described in the “Control intervention” paragraph, e.g., Functional Electrical Stimulation, neuromodulation, or other approaches will be permitted.

The randomization sequence will be generated using simple computer-generated randomization, while allocation concealment will be ensured through sequentially numbered, opaque, sealed envelopes, prepared by independent staff and opened in order at the time of enrollment. Smooth enrollment will be ensured through early patient identification, transparent information and privacy procedures, sustained engagement of clinical staff, and recruitment across multiple sites.

### 2.6 Experimental intervention

ROOMMATE I. In phase 2, the VRRS1 will be enriched with digital educational and rehabilitation contents developed by a multidisciplinary team, following the collection of stroke stakeholders’ needs. The digital content will provide general knowledge about the pathogenesis and prognosis of stroke, and strategies and advice for self-management and/or caregiver-assisted management of the condition. It will also include practical information about the daily therapy scheduling and profiles of all health professionals involved in patient care. The rehabilitation content will include system-guided motor and cognitive exercises that the patient can perform independently or with the help/supervision of their family member or caregiver. The wide range of digital content (pre-existing VRRS features and new educational/rehabilitation content) will be customized by the therapists, based on the specific rehabilitation objectives.

ROOMMATE II. In phase 3, the VRR1 will be integrated with the BM4ROOMMATE system, developed by the University of Florence to provide and integrated system VRRS2. The BM4ROOMMATE is composed of (i) a set of Inertial Measurement Units (IMUs) that should be placed on the hand. (ii) a custom interface to manage the data acquisition and (iii) custom algorithms to measure patients’ performance on a selected set of exercises (e.g., velocity, IAV, number of repetitions). BMR4ROOMMATE is connected through Bluetooth to Khymeia platform so this data can be also visualized from the clinician, if necessary. The measures derived from BMR4ROOMMATE will be used to monitor motor recovery and to assist clinicians in evaluating and objectifying motor performance. All end users will have access 7 days a week to the system’s functionalities and digital contents. The therapists will indicate to the patients and/or their families the most suitable content according to patients’ clinical status. The e-HC will be available in person 5 days a week to train stroke survivors and caregivers and empower them to make independent use of the proposed technologies. In each phase, the intervention will last 3 weeks.

The description of the intervention according to the Template for Intervention Description and Replication (TIDIER) (22) checklist is provided in Supplementary material.

### 2.7 Control intervention

All participants will receive UC, meaning they will be involved in the usual stroke rehabilitation pathway, undergoing the individual rehabilitation project, personalized based on needs and adapted according to the severity of the deficits, energy reserve, degree of frailty, and the main objectives of the rehabilitation program. The UC pathway has been developed in reference to the American Heart/America Stroke Association (AHA/ASA) (4), European Stroke Organization (23), and Italian stroke prevention and educational awareness diffusion (SPREAD) guidelines (24). Both guidelines emphasize intensive interdisciplinary rehabilitation and the Italian guideline specifies that this means at least 3 h of specific rehabilitation per day, including all specific motor and cognitive interventions. UC includes physiotherapy, OT interventions for activities of daily living (ADL) training, pharmacological interventions and nursing care, as well as, according to individual needs, cognitive assessment and training, deglutition, speech and language assessment and training, and psychological support to patient and family. Care is individually tailored to patient’s needs and to the type and severity degree of the identified functional deficits and activity limitations, and to the objectives of the rehabilitation program. Including comorbidities, also intervening on risk factors to prevent complications, recurrences or onset of new diseases.

### 2.8 Outcome and assessment points

Participants will undergo a baseline (T0) and a post-treatment (T1) multidimensional assessment based on the Minimal Assessment Protocol for Stroke rehabilitation (PMIC2020) developed by the Stroke Section of the Italian Physical and Rehabilitation Medicine Association (25). PMIC2020 will be integrated with recommended in-depth assessment of some dimensions such as motricity (26) and cognitive status (27). At T0, demographic (sex, age, education) and clinical (type of stroke, time since stroke, lesion laterality) variables will also be collected in addition to primary and secondary clinical outcomes, whereas T1 assessment will include all primary and secondary clinical outcomes and user experience, usability, feasibility and social impact measures.

For both RCTs, the primary outcome will be the independence in basic daily activities, measured by the modified Barthel Index (mBI) with a score 0–100, because it is a clinically meaningful goal for patients, healthcare professionals and healthcare systems, to which various rehabilitation interventions contribute.

- Primary outcome measure: the modified Barthel Index (mBI) was selected to assess the level of disability, using a score from 0 to 100 (28).
- Secondary clinical outcome measures:
  - Motor outcomes:
    - to measure manual dexterity the 9-Hole Peg Test (9-HPT) was selected, with a score based on the total number of seconds taken by the subject to complete the test (29)
    - the Fugl-Meyer Assessment scale, upper-limb section (FMA-UL), which evaluates senso-motor recovery with a score ranging 0–66 (26, 30);
    - the Action Research Arm Test (ARAT) which evaluates upper limb function, with a score from 0 to 57 points (31).
    - the Motricity Index (MI) which evaluates upper and lower limb muscle strength with a score from 0 to 100 (32).
    - the Functional Ambulation Category (FAC) which evaluates the level of independence in ambulation with a score from 0 to 5 (33)
    - Sensors based metrics of motor performance of the upper limb, derived both from Khymeia and from DIEF sensors (only in ROMMATE 2^nd^).
  - Cognitive outcomes:
    - Montreal Cognitive Assessment (MoCA) which evaluates cognitive function with score from 0 to 30 (27);
    - the Hearth Test (HT) of the Oxford Cognitive Screen (OCS) which evaluates allocentric and egocentric neglect (34)
    - the Hospital Anxiety and Depression Scale (HADS), which evaluates anxiety and depression using a score 0–21 (35).
  - Quality of life:
    - the Short Form-12 (SF-12), which evaluates health-related quality of life with a score range from 0 to 100 (36).

Additional secondary outcomes will be related to user experience, usability and feasibility, which will provide insight into the safety, acceptability, and scalability of the intervention, essential elements for real-world implementation:

- User’s experience:
  - the Training Evaluation Inventory (TEI) which evaluates the effectiveness of the training to the use of technology (37);
  - the Unified theory of acceptance and use of technology (UTAUT) and the Digital Adoption Questionnaire which evaluate the perceived usefulness, perceived ease of use, and user acceptance (38, 39);
  - Numerical Rating Scale (NRS 0–10) which evaluates the overall patient and caregiver satisfaction with the rehabilitation experience.
- Usability:
  - the eHealth UsaBility Benchmarking Instrument (HUBBI) which evaluates the usability of products (40)
  - the Wearability questionnaire which evaluates the user experience and satisfaction with BM4ROOMMATE system (only in ROOMMATE 2^nd^) (41).
- Feasibility:
  - The number and kind of side effects and technology failure will be recorded to assess the feasibility of the VRRS supported treatment.

In cases where patients will not be able to use the system independently, the user’s experience and usability outcomes will be also assessed by their caregivers.

### 2.9 Sample size estimation and data analysis

We estimated the sample size for the first RCT (ROOMMATE 1, phase 2) based on the primary outcome (mBI). A previous RCT (13) tested the effectiveness of an enriched environmental activities program compared to UC in an inpatient neurorehabilitation unit and found an effect size equal to about 0.25, as measured by the motor section of the Functional Independence Measure. This scale assesses the same domain as the mBI (i.e., independence in basic ADL). We hypothesize that the intervention proposed in ROOMMATE might have a larger effect, since we will develop ad-hoc infotainments and rehabilitation contents that will be available bedside throughout the day and that will involve informal and formal caregivers as well. Thus, considering a medium effect size (0.5), a power of 0.80 and an alpha error of 0.05, we estimated a minimum sample size equal to 64 for each group, increased to 70 to account for a 10% drop-out rate. In the second RCT we planned to enroll 60 patients in total in the two participating clinical centres, an adequate sample for pilot studies according to Pearson et al., 2020 (42).

The Shapiro–Wilk test (*p* < 0.05) will be preliminarily used to check the data distribution. Continuous variables will be summarize as mean (SD) or median [IQR], as appropriate, and categorical variables as frequencies or percentages. Primary analysis metric is the difference in change from baseline between groups. For all variables, parametric (repeated measures 2 × 2 ANOVA) or non-parametric (Mann–Whitney test, Wilcoxon test) statistics will be used depending on the distribution of the data to detect within- and between-group differences over time. In addition, the proportion of participants showing changes greater than the estimated MCID for each outcome measure, when available, will be analysed using the chi squared test. All analyses will follow the intention-to-treat principle. Significance will be set at *p* < 0.05 in all analyses.

### 2.10 Monitoring plan and data management in the clinical investigation

This study will be internally monitored by the Principal Investigators and the Scientific Coordinator through weekly formal meetings to assess recruitment progress and data integrity, implementing corrective actions when necessary. Outcome and baseline data will be collected following the Minimal Assessment Protocol for Stroke rehabilitation (PMIC2020), using standardized and validated instruments (e.g., modified Barthel Index, Fugl-Meyer, ARAT, MoCA, HADS, SF-12), administered by trained assessors to ensure reliability and consistency. Data will be collected using both paper and electronic tools, pseudonymized, and securely stored in the RedCap platform. Aggregated data and indicators derived from RedCap will also be transferred to the SMILEPlatform, a dedicated dashboard for socio-economic and digital adoption Key Performance Indicators (KPIs). Access will be restricted to designated investigators, ensuring compliance with General Data Protection Regulation (GDPR) regulations. Data quality will be promoted through range checks, monthly audits, duplicate entry for selected variables, and continuous training of assessors. To promote adherence and reduce dropouts, patients and caregivers will be supported by trained e-Health Coaches, who will provide continuous guidance on the use of the digital ecosystem, promptly address technical malfunctions, and encourage regular engagement with the intervention. Data will be retained for 10 years and analyzed collectively to evaluate the impact of the intervention. In case of participant withdrawal or protocol deviation, available outcome data (including those retrievable from clinical records) will still be collected to minimize attrition bias. Adverse events will be reported following EU Regulation 2017/745 and Medical Device Coordination Group (MDCG) guidelines. Given the nature of the study, potential adverse effects are minimal, primarily related to patient frustration or screen fatigue, which will be mitigated through support strategies. All statistical analyses will follow the intention-to-treat principle, with sensitivity analyses as appropriate, and missing data will be managed using standard methods, including multiple imputations where applicable. No formal Data Monitoring Committee is foreseen given the low-risk profile of the intervention. Monitoring is instead ensured by the Consortium governance structure (Steering Committee and External Advisory Board) as established in the Consortium Agreement. Interim analyses are not planned, since the trial focuses on short-term outcomes, but recruitment, data integrity, and safety will be periodically reviewed in internal meetings. Adverse events will be documented in RedCap and reported according to EU MDR guidelines. Auditing is embedded within the governance structure, with traceability assured through meeting minutes, audit trails, and dataset versioning.

### 2.11 Ethics and dissemination

The study protocol has received approval from the competent Ethics Committees at all recruiting sites before patient enrolment. Any protocol amendments will be formally submitted for Ethics Committee review and communicated to all partners according to procedures defined in the Consortium Agreement. Written informed consent will be obtained from all participants (or legal representatives when necessary), using harmonized templates adapted to national languages and settings, in line with GDPR requirements. The English version of the informed consent form is provided in the study appendices. Confidentiality will be ensured through pseudonymisation of all personal data, restricted access to databases, and compliance with data protection procedures described in the Data Management Plan. Investigators will declare the absence of financial or commercial interests related to the devices under study. Access to data will be restricted to authorized consortium members; de-identified aggregated data may be shared in open repositories (e.g., Zenodo) following the principle “as open as possible, as closed as necessary”. Dissemination of results will follow the policy set out in the Consortium Agreement, including prior notification of partners, protection of confidential information, and open-access publication of scientific outputs. Results will also be communicated to patients, caregivers, and healthcare professionals through dedicated dissemination activities.

## 3 Discussion

Over the past decade, significant progress has been made in the treatment of subacute stroke. However, stroke remains a major cause of disability worldwide. While rehabilitation can significantly alleviate post-stroke disability and socio-economic burden, there remain several research challenges, particularly in personalizing rehabilitation strategies and developing novel outcomes that overcome current limitations. The optimization of these rehabilitation pathways processes is still hindered by organizational and cultural barriers to resources allocation and innovation adoption (3).

A main reference document for the outcome definition was the PMIC2020 (25), an updated version of the PMIC (43), the minimal protocol to assess stroke survivors. Among the others, the PMIC2020 identifies the mBI (28) as the reference measure of functioning after stroke. Indeed, the modified version of the Barthel Index (BI) was originally developed with the aim of improving the sensitivity and the reliability of the original tool, showing this advantage. As well, Wang et al., 2022 (44), found the mBI to have a better responsiveness than the BI at both the group and individual levels in the patients with early subacute stroke.

While functional independence remains the primary goal for stroke survivors, around 70% of them develop sensorimotor impairments, with only a small proportion recovering within 6 months after the stroke. To address the need for a comprehensive assessment of hand and upper limb functions, we have integrated a series of clinical and sensors-derived measures in ROOMMATE aimed at focusing on the assessment of these functions. Specifically, among the technologies envisaged in the ROOMMATE project, we selected the BMR4ROOMMATE wearable device for monitoring hand and finger motor functions that will be used in the 3^rd^ phase of the study.

As clinical measures of the upper limb functions, we have chosen the three outcome measures that were recommended by a modified Delphi process by the Stroke Taskforce (StrokEDGE), a panel of research and clinical experts. The Fugl-Meyer Upper Limb section, as it is a reliable (45), valid (46) and responsive (47) measure of the upper-limb sensory-motor recovery, validated both in Italian (48, 49) and in Romanian (50). The 9-HPT is a standardized, quantitative assessment used to measure finger dexterity, also recommended as a more in-depth assessment in PMIC2020. The ARAT was chosen to assess the overall upper limb functional performance, according to recommendations of the Stroke Rehabilitation Round Table. This outcome showed excellent to adequate metric properties on stroke survivors (51–53).

Also, for cognitive assessment, we chose the MoCA as it is recommended also in the PMIC for research purposes. Indeed, the MoCA requires longer time for administration and trained examiners, but has higher sensitivity than the Mini Mental State Examination and executive functions (27). This test allows us to assess several cognitive abilities at once: constructional praxis, executive function, visual–spatial function, attention, verbal comprehension and working memory (27). To assess the allocentric and egocentric neglect it was decided also to include the HT of the OCS.

The HADS was included in the protocol because mood disorders are significant effects of stroke, impacting both recovery and overall outcomes while also adding to caregiver stress (54–57). Despite the fact that anxiety and depression are frequently observed after a stroke, with anxiety affecting 14–28% and depression affecting 11–61% of patients, these conditions are often not recognized and insufficiently treated (57–61). Our hypothesis is that the digital ecosystem provided in the ROOMMATE phases along with the dedicated coaching may significantly impact on depression and anxiety of stroke patients involved in post-acute inpatient rehabilitation.

The same time, we hypothesize that this intervention may impact on the patients’ quality of life, measured by the SF-12 was created to replicate the physical and mental summary scores of the short form-36 (SF-36) (36), and research has shown a strong correlation between the summary scores generated by the SF-12 and SF-36, particularly in populations such as stroke survivors (62). Most RCT towards the effectiveness and safety of new rehabilitation pathway supported by technology, such as ROOMMATE focus on a single perspective (63). To ensure sustainable implementation of ROOMMATE we focus on the end-user, clinical and societal perspective as recommended by Jansen-Kosterink et al., 2022 (64). Concerning the end-user perspective, it is also known that incorporating users’ experience measurements into rehabilitation protocols is essential for tailoring interventions to individual needs, enhancing engagement, and improving outcomes. By prioritizing user feedback, practitioners can better understand barriers to progress and refine strategies to optimize rehabilitation effectiveness. As described above, the clinical perspective is addressed by means of multiple questionnaires based on guidelines and earlier research. To properly assess the end-user perspective, we added a questionnaire focusing on this domain. These questionnaires are based on technology acceptance model (TAM), the most common model used in research to assess technology acceptance. By focusing on users’ perceptions of ease of use and usefulness, TAM provides insights into the factors that drive technology acceptance and informs strategies to enhance user engagement and satisfaction (39, 65). Next to acceptance, also the usability and wearability of the designed technologies will be researched. Including a usability and wearability scale in a rehabilitation protocol is important for a user-centered approach design, to identify barriers to the exploitation of a product/ service and get feedback to improve them. Although the System Usability Scale is one of the most used scales to assess usability, in the current research authors decided to use the HUBBI scale (40). SUS indeed tends to be quite generic and does not consider the unique features of e-health applications. To address this gap, the HUBBI was created as a new, comprehensive usability benchmarking tool tailored for the eHealth sector (66).

Based on the outcomes of this cmRCT we will also assess the societal impact of ROOMATE (societal perspective), by calculation an Social Return on Investment (SROI) ratio as described by the social return on investment (SROI) method (67). This method is described as “a framework for measuring and accounting for this much broader concept of value; it seeks to reduce inequality and environmental degradation and improve wellbeing by incorporating social, environmental and economic costs and benefits.” Overall, in line with ROOMMATE inclusive perspective, this method looks at the total value including non-monetary costs and benefits (68).

ROOMMATE introduces an innovative approach to stroke rehabilitation by integrating accessible and sustainable digital solutions into clinical pathways. By combining sensor-based assessment, expert coaching on digital literacy, and the active involvement of patients, caregivers, and healthcare professionals, and by rigorously verifying its impact by a cmRCT, it seeks evidence to enhance rehabilitation intensity and optimize resource utilization. The ecosystem promotes a people-centred approach, facilitating the adoption of evidence-based digital tools while addressing key challenges such as accessibility, equity, and sustainability. Through interdisciplinary collaboration and technology-supported clinical pathways, ROOMMATE has the potential to improve patient outcomes, enhance the efficiency of healthcare services, and support the broader adoption of digital health solutions in rehabilitation.

Limitations of our study are mostly due to time, i.e., the short duration of treatment, set at 3 weeks. Such a short duration was a necessary choice, since the participants will be recruited among patients admitted for post-stroke in-patient rehabilitation, and the duration of the training is therefore conditioned by the length of hospitalization. While this period is the average time assigned to patients for rehabilitation stays, it may still be insufficient to achieve significant results or to fully assess the effectiveness of the implemented rehabilitation strategies. Further the diversified needs and the personalized contents of the interventions may produce diverse effects, not always best captured by our necessarily general primary outcome (ADL disability as measured by the MBI). Additionally, the brief duration limits our ability to monitor patients’ long-term progress, reducing our understanding of the potential long-term effects of the treatment. Further development of the ROOMMATE project will be necessary to adequately address these issues, including a follow-up in the next experiments.

In conclusion, the ROOMMATE will rigorously verify the short-term impact of a digital ecosystem in sub-acute post-stroke rehabilitation, compared to UC, co-designed in collaboration with patients, their families, patient associations, and healthcare professionals. The strength of this study lies in its inclusive approach, involving all stakeholders to provide diverse perspectives and define precise user and system requirements, which will be integrated into the innovative therapeutic system, and to innovative cmRCT study design, allowing them to overcome many ethical and logistic issues of standard pragmatic RCTs. Authors expect that this human-centered approach will position ROOMMATE at the forefront of stroke care.
