# Supplementary material for: Development of a cohort multiple randomized clinical trial to test an integrated system of sensors and multimedia monitors technology, for stroke rehabilitation: the ROOMMATE study protocol
Source: Front Neurol. 2025 Oct 14;16:1568728. doi: 10.3389/fneur.2025.1568728 (PMC12558804; doi:10.3389/fneur.2025.1568728)
Supplement: Supplementary file 1 [file Data_Sheet_1.PDF]

# INFORMATION FOR THE PATIENT

Version 3.0 (04/03/2025)

**Study Title:** Aggregated system of sensors and multimedia monitors: technology for innovation and personalization of rehabilitation care. (ROOMMATE – AggRegated system Of sensOrs and Multimedia Monitors: technology for innovAtion and personalizaTion of rEhabilitation care)

**Protocol code, version and date:** ROOMMATE Version 3.0 (04/03/2025)

**Study Sponsor:** IRCCS Fondazione Don Carlo Gnocchi

**Scientific Coordinator:** Prof. Francesca Cecchi, fcecchi@dongnocchi.it, IRCCS Fondazione Don Carlo Gnocchi, Florence

**Principal Investigator, Clinical Site:** Prof. Francesca Cecchi, IRCCS Fondazione Don Carlo Gnocchi, Florence

Dear Madam / Dear Sir,

You have been asked to participate in a clinical study and this document has the purpose of informing you about the nature of the study, the aim it intends to achieve, what such participation will entail for you, your rights and your responsibilities.

Please read carefully this written information before making a decision regarding your possible participation in the study. You will have all the time you need to decide whether or not to participate.

Furthermore, you may freely ask any clarification questions and raise again any issue that has not received a clear and exhaustive answer.

In case, after having read and understood all the information provided herein, you decide that you want to participate in the clinical study, you will be asked to sign and personally date the Informed Consent Form attached to this document.

Your personal data will be processed as described in the specific information sheet on the processing of personal data, in compliance with Regulation EU 2016/679 General Regulation concerning the protection of natural persons with regard to the processing of personal data, as well as the free circulation of such data… and Legislative Decree 30 June 2003 Code concerning the protection of personal data containing provisions for the adaptation of the national legal system to Regulation (EU) n. 2016/679; such information and the related request for authorization to the processing of data will be submitted to you separately.

**What the study aims to do**

The study has the objective of monitoring functional recovery, after conventional treatment, in people who have suffered a cerebral stroke.

**What are the characteristics of this study**

This is an observational study conducted at IRCCS Fondazione Don Carlo Gnocchi in Florence, Italy. The study will enroll 100 people with subacute stroke outcomes for 13 months from the approval of the ethics committee. The study foresees the functional and cognitive evaluation of all subjects who agree to participate, at the beginning of the hospitalization period and at the end of the hospitalization period in hospital. The subjects will undergo the conventional treatment provided by the clinical inpatient center. The study foresees subsequent phases. Each subject may be asked to participate in subsequent phases without knowing which phase it is. The possible participation in subsequent phases of the study will be bound to the acceptance by the subject of the information consent form for participation in the study and of the information form for the processing of specific personal data.

Participation in the study will have a duration of about 3 weeks and at least 100 patients will be enrolled at this Hospital, IRCCS Fondazione Don Carlo Gnocchi of Florence.

**3. What your participation in the study entails**

In the event that you decide to participate in the study, we inform you that, after having assessed the possibility of being able to include you in the research and after having carried out all the medical/instrumental treatments foreseen for you, regardless of your participation in this research, which will not affect the patient's therapeutic pathway. The study foresees a functional and cognitive evaluation at the beginning and at the end of the hospitalization period at the clinical center IRCCS Fondazione Don Carlo Gnocchi of Florence, with an interval of 3 weeks between the two evaluations. During this period, the patient will benefit from the conventional treatment reserved for all inpatients of the center.

**4. What are the benefits you may receive by participating in the study**

Although this cannot be guaranteed in advance, there is no direct benefit from participating in the study.

**5. What happens if you suffer an injury during the research study?**

We inform you that the Sponsor has taken out, as required by the Ministerial Decree 14 July 2009, an insurance policy with Lloyd's insurance Company S.A. which guarantees specific coverage for the compensation of damages caused to subjects by the experimentation activity, for the entire period of the same, covering the civil liability of the investigator and the sponsor, without excluding damages unintentionally caused as a consequence of an accidental event and/or attributable to negligence, imprudence or inexperience. This insurance coverage guarantees a maximum damage compensation of €1,000,000.00 per patient, with a limit of €7,500,000.00 per protocol. The policy is operative exclusively for damages that have manifested no later than 36 months from the end of the trial for which a compensation request has been submitted by the conclusion date of the trial. Exceeding the above limits and the previous restrictions do not prejudice your right to request any compensation directly from the person responsible for the damage. By signing this informed consent you do not waive any of your legal rights. Before joining the trial, if you have taken out

an insurance policy, it is advisable that you verify with your insurer that your participation does not have any impact on it. The above insurance policy provides for the following exclusions from coverage (list follows with transcription of all excluded guarantees):

a) for trials not regularly authorized and/or started without proper information and consent and/or intentionally conducted in a manner different from that authorized by the Competent Authorities and/or in the absence of patient signed informed consent;
b) for damages that are not causally related, in the terms established by the applicable Laws and Decrees, with the insured Trial;
c) for claims due to the fact that the product and/or the therapeutic investigation and/or the Medical Device does not achieve the intended curative purposes;
d) for damages to pregnant women, for congenital, genetic damages and/or malformations caused to the fetus;
e) for genetic damages and for genetic and/or hereditary diseases;
f) for claims deriving from the use of systems, machinery and chemical or nuclear substances that are not compliant with the law;
g) for claims due to acquired immunodeficiency from HIV or to incorrect diagnosis of such syndrome;
h) for damages deriving from the use of surgical activities.

**6. Possible alternatives**

The study does not differ from and does not affect normal clinical practice.

**7. What happens if you decide not to participate in the study**

Participation in the study is completely voluntary: you are free not to participate in the study or, if you decide to participate, you will have the right to withdraw from the study at any time and without the obligation to provide explanations, however giving notice to the study doctor, Prof. Francesca Cecchi. In such case no further data concerning you will be collected and you may request the deletion of those already collected. Your current and future medical care at the IRCCS Fondazione Don Carlo Gnocchi Hospital in Florence will not be compromised by your decision and the doctors will continue to follow you with due care.

On the other hand, your participation in the study may be interrupted if the doctor assesses that the new treatment has brought you no benefit or if adverse effects occur. In these cases you will be promptly informed by the doctor and you may discuss with him further valid treatments for your disease.

**8. Procedures foreseen at the end of the study**

The procedures foreseen at the end of the study will not differ from normal clinical practice.

**9. Consent to inform your general practitioner**

For the best protection of your health, you will be asked to inform your general practitioner about the trial you agree to participate in: his/her involvement is very important in order to

avoid possible harm resulting from the prescription/intake of drugs or other products that could interact, even seriously, with the treatments foreseen by the study.

**10. Information about the results of the study**

If you request it, at the end of the study you may be informed of the general results of the study and in particular those concerning you.

A description of this clinical trial will be available on the website http://www.ClinicalTrials.gov.

This/these website(s) will not contain information that can identify you. At most, the website will include a summary of the results. You can consult the website at any time. Please note that this site is available only in English and therefore, if necessary, you may request the assistance of the study doctor to access the desired information.

**11. Further information**

No additional costs will be charged to you due to participation in the study.

You will not receive any financial compensation for participation in the study. The study protocol proposed to you has been approved by the Ethics Committee ________________ on date _____________. The Ethics Committee has, among other things, verified the compliance of the study with the European Union Good Clinical Practice Guidelines and the ethical principles expressed in the Declaration of Helsinki.

You may report any matter you deem appropriate to highlight, regarding the research that concerns you, to the Ethics Committee and/or the Health Directorate of this hospital facility.

In case you have questions about the study or about participation in this study, in case you think you have suffered harm related to the study, in case you have questions about your rights as a participant, you must contact Dr. _______________ at number _____________________, email ______________________.

## INFORMED CONSENT FORM FOR PARTICIPATION IN THE STUDY

**Study Title:** Aggregated system of sensors and multimedia monitors: technology for innovation and personalization of rehabilitation care. (ROOMMATE – AggRegated system Of sensOrs and Multimedia Monitors: technology for innovAtion and personalizaTion of rEhabilitation care)

**Protocol code, version and date:** ROOMMATE Version 3.0 (04/03/2025)

**Study Sponsor:** IRCCS Fondazione Don Carlo Gnocchi

**Scientific Coordinator:** Prof. Francesca Cecchi, fcecchi@dongnocchi.it, IRCCS Fondazione Don Carlo Gnocchi of Florence

**Principal Investigator, Clinical Site:** Prof. Francesca Cecchi, IRCCS Fondazione Don Carlo Gnocchi of Florence

I, the undersigned ___________________________________________ born on //_____ resident in ____________________ street/square __________________________ Tel.______________ domicile (if different from residence) ________________________________

DECLARE

that I have received from Dr. ______________________________________ exhaustive explanations regarding the request to participate in the research in question, according to what is reported in the information sheet, which is part of this consent, of which a copy was given to me on date ___________ at time _____________ (indicate date and time of delivery);

that I have read the patient information sheet related to this study, that I am aware that such participation is completely voluntary and that I agree to follow the instructions of the study doctor;

that the nature, the purposes, the procedures, the expected benefits, the possible risks and inconveniences and the alternatives of the clinical study have been clearly explained to me and I have understood them;

that I had the opportunity to ask clarifying questions and received satisfactory answers;

that I had all the necessary time before deciding whether or not to participate;

that I have not been subject to any undue coercion in the request for Consent;

that it has been clearly explained to me that I can freely decide not to take part in the study or to withdraw at any time without providing justification and without penalties, and that such decisions will in no way affect the relationship with my treating doctors and with the institution where I am being treated;

that I am aware of the importance (and of my responsibility) of informing my general practitioner about the trial I agree to participate in; if I decide not to inform him/her, I release both my general practitioner and the doctors following me in the trial from responsibility for any damage that may arise from incompatibility between the study drug(s) and other medical treatments;

that I am aware that I have the right to request a dated and signed copy of this informed consent form.

Therefore I DECLARE that I:

☐ want ☐ DO NOT want to participate in the study

☐ want ☐ DO NOT want to be informed of the results of this research by the study doctor

☐ want ☐ DO NOT want to be informed of the results of the research by the study doctor, also in relation to unexpected findings that may be accidentally discovered with the investigations foreseen by the study

☐ want ☐ DO NOT want my general practitioner to be informed of participation in the study

__________________________________ ______ _________ _____________________

Full name of patient Date Time Signature
(Adult or from the age of 16)

__________________________________ ______ _________ ____________________

Full name of legal representative Date Time Signature

If the patient, or the recognized legal representative, is unable to read:

I participated in the entire discussion for informed consent. I certify that the information in the consent form or any other written information has been accurately explained and apparently understood by the patient or the patient's recognized legal representative. Informed consent was freely given by the patient or the patient's recognized legal representative.

___________________________ ______ _____________ _________________

Full name Date Time Signature
of the impartial witness

I, the undersigned Prof./Dr.

......................................
................................

Surname
Name

Declare that the Patient voluntarily signed his/her participation in the study

I also declare that:

I provided the Patient with exhaustive explanations regarding the purposes of the study, the procedures, the possible risks and benefits and its possible alternatives;
I verified that the Patient sufficiently understood the information provided;
I gave the Patient the necessary time and the possibility to ask questions about the study;
I did not exercise any coercion or undue influence in the request for Consent.

________________________ ______ _________ ____________________

Full name of the physician Date Time Signature
who provided the information and
collected the informed consent

**INFORMATION ON THE PROCESSING OF PERSONAL DATA**

Pursuant to the EU General Data Protection Regulation 2016/679 and Legislative Decree 196/2003

**Study Title:** Aggregated system of sensors and multimedia monitors: technology for innovation and personalization of rehabilitation care. (ROOMMATE – AggRegated system Of sensOrs and Multimedia Monitors: technology for innovAtion and personalizaTion of rEhabilitation care)

**Protocol code, version and date:** ROOMMATE Version 3.0 (04/03/2025)

**Study Sponsor:** IRCCS Fondazione Don Carlo Gnocchi

**Scientific Coordinator:** Prof. Francesca Cecchi, fcecchi@dongnocchi.it, IRCCS Fondazione Don Carlo Gnocchi of Florence

**Principal Investigator, Clinical Site:** Prof. Francesca Cecchi, fcecchi@dongnocchi.it, IRCCS Fondazione Don Carlo Gnocchi of Florence

**Purpose of the processing**

The center where the study is conducted is the IRCCS Fondazione Don Carlo Gnocchi of Florence, which commissioned the study described to you. The study will be conducted in compliance with the responsibilities provided by the Good Clinical Practice guidelines (Legislative Decree no. 211/2003), Clinical Trials Regulation, EU Regulation 2016/679 (GDPR) and Legislative Decree 30 June 2003 Data Protection Code ... Your personal data will be processed, as specified below, exclusively insofar as they are essential for the conduct of the study and the achievement of its objectives. For any further purposes of future clinical research, you will be presented with specific documentation and the related information notice.

The processing concerns personal data (art. 4 n. 1 GDPR), including those falling under the categories of art. 9 GDPR; in particular: age, sex, education and diagnosis.

**Provision and nature of processed data**

In general, only the staff of the Center where the study is conducted (for example, the study doctor and/or nurse) will have direct access to your personal data. However, the Sponsor is legally required to appoint a study supervisor who will have access to your personal data, but only to verify the quality of the data collected for the study. In addition, it may also happen that members of the Ethics Committee and representatives of national or international public authorities are authorized to access your personal data, if required by the applicable law.

The provision of personal data for the clinical study is indispensable for the conduct of the study and refusal to provide them will not allow you to participate.

**Methods of processing. Dissemination and communication of data.**

The data will be processed by paper and electronic tools accessible only to the investigators involved in the research. With the prior consent of the subject or his/her family

member/caregiver/legal guardian, the data will be entered in pseudonymized form into a computerized database using REDCap software (Research Electronic Data Capture; http://redcap.dongnocchi.it/).

Your participation in the study implies that, in compliance with clinical trial regulations, Sponsor personnel or external companies that carry out monitoring and verification of the study on behalf of the Sponsor, the Ethics Committee and the Italian and foreign Health Authorities may access the data contained in your original clinical documentation, in ways that guarantee the confidentiality of your identity.

The study center where the study is conducted will adopt all the necessary security measures and appropriate technical safeguards to carry out processing in compliance with current legislation and to protect your personal data, your dignity and confidentiality.

For the purposes indicated above, your personal data will be collected by the study center (through its authorized staff, such as the study doctor and/or nurse) and transferred in pseudonymized form, by electronic or other means, to the following recipients:

- The Sponsor of the study;
- The Sponsor's affiliates;
- Italian regulatory authorities (e.g., Italian Ministry of Health), European and non-European authorities, the Ethics Committee and the specific recipients of notifications under the law.

**Pseudonymisation of data**

The pseudonymisation of personal data means that your personal data are processed in such a way that they can no longer be attributed to you without the use of additional information, provided that such additional information is kept separately and subject to technical and organizational measures. In other words, the study center will identify you with an identification code at the time of your involvement in the study. This identification code will be used by the Center instead of your name in each communication to the Sponsor of data connected to the study. The Sponsor will record, process and store your personal data, as well as any other data collected concerning this study, together with your identification code. The study center will be the only and exclusive entity able to associate the identification code with your personal data. Furthermore, such code will be kept in confidential documents and will be accessible only when indispensable for the purposes of the study and for limited periods of time (e.g., during monitoring and verification activities).

The Sponsor's affiliates and persons or external companies operating on behalf of the Sponsor will process personal data as data processors or third parties authorized to process personal data, depending on the specific situation and in compliance with the applicable privacy law. The complete and updated list of parties appointed as data processors and of

third parties authorized to process personal data is available by contacting the following email address: fcecchi@dongnocchi.it.

The data will be owned by the clinical center as data controller pursuant to art. 26 of EU Regulation 2016/679, in relation to the processing of personal data connected to ROOMMATE. The purposes of the processing are to evaluate and improve the functional recovery of people with subacute stroke outcomes through the implementation of an advanced bedside technological solution and the provision of dedicated coaching.

The Sponsor will stipulate a separate data protection agreement with each recipient of your pseudonymized data acting on behalf of the Sponsor to carry out activities functional to the realization of the scientific study, in order to guarantee that the data are processed in compliance with this information notice.

In the event that your pseudonymized data are transferred to a recipient located in a country outside the European Union, the Sponsor will ensure that the cross-border processing of data is adequately protected and conducted only on the basis of standard data processing clauses that comply with the GDPR and all further requirements defined by the applicable laws.

**Data retention**

Your pseudonymized data will be kept for a period not exceeding that necessary for the purposes of the study for which they were collected – at least 10 (ten) years from the conclusion of the study – or for a longer period if required by the contractual agreement between the Sponsor and the study centers, always in accordance with the mandatory retention periods defined by the applicable laws. Any personal data will be deleted after the expiry of the applicable retention period. In any case, you have the right to request, at any time, the deletion of data, in compliance with the GDPR and applicable data protection law.

**Exercise of rights**

You have the right to access your personal data processed for the study and request their rectification, limitation, or deletion. Furthermore, you may revoke your consent to participation in the study and to the collection of further data at any time.

For these requests, you can contact in writing the study center:

IRCCS Fondazione Don Carlo Gnocchi of Florence
Email: direzione.polotoscana@dongnocchi.it
Data Protection Officer (DPO): rpd@dongnocchi.it
Principal Investigator: Prof. Francesca Cecchi, fcecchi@dongnocchi.it

Without the support of the study center, the Sponsor will not be able to satisfy any of your requests due to the lack of information (e.g., which series of pseudonymized data is related to you). You have the right to withdraw from the study at any time. The Sponsor will be informed by the study doctor of the withdrawal of your consent to participation in the study and no further information about you will be collected. You may also exercise the right to be forgotten

(art. 17 GDPR) and therefore request the deletion of all personal data collected: however, this right may not be fully recognized if the retention of data related to you is necessary to comply with a legal obligation requiring the processing of such data and/or insofar as their deletion would risk making it impossible or seriously prejudicing the achievement of the scientific objectives of this study connected to the processing. You always have the right to lodge a complaint with the supervisory authority (in Italy the Data Protection Authority, www.garanteprivacy.it, email garante@gpdp.it, switchboard tel. 06696771).

**Data controllers and processors**

The Data Controller for healthcare purposes of care, diagnosis and prevention and for the execution of this Study is the study center: Prof. Francesca Cecchi, fcecchi@dongnocchi.it.

For information regarding data processing and related rights, please contact the Data Protection Officer of the study center.

**DECLARATION OF CONSENT TO THE PROCESSING OF PERSONAL DATA**

Pursuant to the EU General Data Protection Regulation 2016/679 and Legislative Decree 196/2003

**Study Title:** Aggregated system of sensors and multimedia monitors: technology for innovation and personalization of rehabilitation care. (ROOMMATE – AggRegated system Of sensOrs and Multimedia Monitors: technology for innovAtion and personalizaTion of rEhabilitation care)

**Protocol code, version and date:** ROOMMATE Version 3.0 (04/03/2025)

**Study Sponsor:** IRCCS Fondazione Don Carlo Gnocchi

**Principal Investigator:** Prof. Francesca Cecchi, fcecchi@dongnocchi.it, IRCCS Fondazione Don Carlo Gnocchi of Florence

---

I, the undersigned ____________________________________________ born on //______ resident in _____________________ street/square ___________________________ Tel.________________ domicile (if different from residence) _________________________________

DECLARE

to explicitly understand and accept that my personal data will be processed for this study by the parties and in the ways described in detail in the Information on the processing of personal data pursuant to the EU General Data Protection Regulation 2016/679 and Legislative Decree 196/2003;

(if applicable) to explicitly understand and accept that my pseudonymized personal data may also be transferred to a recipient in a country outside Europe (specify the identification details of the recipients), where the General Data Protection Regulation does not apply;

to explicitly understand and accept that any cross-border processing of data outside Europe will be adequately protected and carried out only on the basis of standard data processing clauses that comply with the General Data Protection Regulation and all further requirements defined by the applicable laws;

to explicitly understand and accept that if I revoke my consent to participation in the study, no further personal data about me will be collected;

to explicitly understand and accept that even after revocation of my consent, the Sponsor will still be authorized to store and process my pseudonymized personal data collected before my revocation exclusively for the purpose of fulfilling legal obligations and/or insofar as such data are essential for the conduct of this study and the achievement of its objectives;

to explicitly understand and accept that for questions regarding data confidentiality in this study I may contact the Data Protection Officer of the study center:

_______________________________, tel. _________________ email:

__________________________

Full name of the Data Protection Officer of the study center

I therefore declare that I give my informed consent to the processing of my personal data, including those falling within the so-called particular categories, for the purposes and in the ways described in the information sheet, which is part of this consent, of which a copy was given to me on date ___________ at time _____________ (indicate date and time of delivery).

_________________________________ ______ _________ ___________________

Full name of patient Date Time Signature
(Adult or from the age of 16)

_________________________________ ______ _________ ___________________

Full name of legal representative Date Time Signature

If the patient, or the recognized legal representative, is unable to read:

I participated in the entire discussion for the informed consent to data processing. I certify that the information in the consent form or any other written information has been accurately explained and apparently understood by the patient or the patient’s recognized legal representative. Informed consent was freely given by the patient or the patient’s recognized legal representative.

__________________________ ______ _____________ ________________

Full name Date Time Signature
of the impartial witness
